# Tomato Sauce Enriched with Olive Oil Exerts Greater Effects on Cardiovascular Disease Risk Factors than Raw Tomato and Tomato Sauce: A Randomized Trial

**DOI:** 10.3390/nu8030170

**Published:** 2016-03-16

**Authors:** Palmira Valderas-Martinez, Gemma Chiva-Blanch, Rosa Casas, Sara Arranz, Miriam Martínez-Huélamo, Mireia Urpi-Sarda, Xavier Torrado, Dolores Corella, Rosa M. Lamuela-Raventós, Ramon Estruch

**Affiliations:** 1 Department of Internal Medicine, Hospital Clínic, Institut d’Investigació Biomèdica August Pi i Sunyer, Medicine School, University of Barcelona, Barcelona 08036, Spain; pvalderas@clinic.ub.es (P.V.-M.); gchiva@clinic.ub.es (G.C.-B.); rcasas1@clinic.ub.es (R.C.); sarranz@clinic.ub.es (S.A.); 2 Biomedical Research Centre in Physiopathology of Obesity and Nutrition (CIBEROBN), Institute of Health Carlos III, Madrid 28029, Spain; mmartinezh@ub.edu (M.M.-H.); dolores.corella@uv.es (D.C.); lamuela@ub.edu (R.M.L.-R.); 3 Department of Nutrition and Food Science, XaRTA, INSA, Pharmacy School, University of Barcelona, Barcelona 08028, Spain; murpi@ub.edu (M.U.-S.); xaviertorrado@ub.edu (X.T.); 4 Department of Preventive Medicine and Public Health, University of Valencia, Valencia 46010, Spain

**Keywords:** tomato, postprandial, cardiovascular, cooked, food matrix, bioavailability

## Abstract

Epidemiological studies have observed a negative association between tomato intake and the incidence of cardiovascular disease. As tomato sauces are usually cooked with the addition of oil, some studies have pointed out that both processes may increase the bioavailability of the bioactive compounds. However, the effect of consumption of raw tomatoes and tomato sauces on inflammation biomarkers and adhesion molecules related to atherosclerosis remains unknown. The aim of this study was to test the postprandial effects of a single dose of raw tomatoes (RT), tomato sauce (TS) and tomato sauce with refined olive oil (TSOO) on cardiovascular disease risk factors. We performed an open, prospective, randomized, cross-over, controlled feeding trial in 40 healthy subjects who randomly received: 7.0 g of RT/kg of body weight (BW), 3.5 g of TS/kg BW, 3.5 g of TSOO/Kg BW and 0.25 g of sugar solved in water/kg BW on a single occasion on four different days. Biochemical parameters and cellular and circulating inflammatory biomarkers were assessed at baseline and 6 h after each intervention. The results indicate that, compared to control intervention, a single tomato intake in any form decreased plasma total cholesterol, triglycerides and several cellular and plasma inflammatory biomarkers, and increased plasma high density lipoproteins (HDL) cholesterol and interleukine (IL) 10 concentrations. However, the changes of plasma IL-6 and vascular cell adhesion molecule-1 (VCAM-1), and lymphocyte function-associated antigen-1 (LFA-1) from T-lymphocytes and CD36 from monocytes were significantly greater after TSOO than after RT and TS interventions. We concluded that tomato intake has beneficial effects on cardiovascular risk factors, especially cooked and enriched with oil.

## 1. Introduction

Cardiovascular diseases (CVD) is the greatest contributor to global morbidity and mortality, according to the World Health Organization (http://www.who.int). The causes of cardiovascular diseases are diverse but the most common is atherosclerosis, a low-grade inflammatory disease that starts with the recruitment of circulating leukocytes and their adhesion to the vascular endothelium [1]. During these cell adhesion processes, endothelial cells are activated by inflammatory cytokines, enhancing the expression of adhesion molecules and synthesizing chemokines and lipid chemoattractants that are presented on their luminal surface [2]. This process, known as endothelial activation, is a key event in the onset of atherosclerosis [3]. Compelling experimental data support the protective role of tomato ingredients against the processes involved in atherogenesis such as endothelial activation [4,5].

Tomato (*Lycopersicon esculentum L*.) is one of the most popular and extensively consumed vegetable crops worldwide [6], and may be consumed either fresh or as tomato processed products (sauce, juice, paste, puree, ketchup and soup). Tomato products are usually cooked with the addition of oil, and both cooking and the addition of a fatty matrix increase the bioavailability of their bioactive compounds [7]. Thus, food matrixes may play a key role in determining the absorption, distribution and final biological action of tomato compounds in the human body [8].

Tomato and its byproducts are rich in phytochemicals such as carotenoids (mainly lycopene and β-carotene), phenolic compounds (mainly flavonoids, such as naringenin), vitamins C and E, potassium and folates [9,10]. Previous epidemiological studies have suggested that tomato intake may decrease the risk of CVD and several cancers [11] due to some of these compounds, mainly lycopene [12]. In this way, tomato consumption could reduce or delay the development of CVD by inhibiting cholesterol synthesis, improving immune function and reducing inflammation [13].

Previous studies have observed that tomato processing may influence the metabolism of tomato phenolics and, consequently, their plasma bioavailability and urinary excretion [14,15,16,17]. However, the effect of acute intake of raw tomato and tomato sauces on plasma and cellular inflammation biomarkers related to the atherosclerotic process remains unknown. We embarked, therefore, on a randomized, controlled feeding intervention trial in order to evaluate the postprandial effects of a single dose of raw tomato (RT), tomato sauce cooked without oil (TS) and tomato sauce cooked with refined olive oil (TSOO) on markers of inflammation and cardiovascular disease.

## 2. Experimental Section

### 2.1. Participants

Forty healthy volunteers were included in the study. None reported any history of CVD or other medical conditions. All subjects were non-smokers and were not receiving medication or vitamin supplements. The Ethics Committee of Clinical Investigation of the University of Barcelona (Spain) and the Institutional Review Board of the Hospital Clinic of Barcelona approved the study protocol. All the participants gave written consent before entering into the trial. The International Standard Randomized Controlled Trial Number of the trial is ISRCTN20409295 (www.controlled-trials.com).

### 2.2. Study Design

The study was an open, prospective, randomized, crossover, controlled feeding trial (Figure S1, Supplementary Material). All subjects underwent a three-day washout period in which they were asked to consume their regular diet, but avoiding the intake of any tomato or tomato-based products and, during the 24 h immediately preceding the dietary intervention as well as the day of the study they were asked to follow a polyphenol-free diet. Subjects received a list showing permitted and forbidden foods and two menus in order to help them to follow the diet correctly.

Each volunteer randomly received: 7.0 g of RT/kg of body weight (BW), 3.5 g of TS/kg BW or 3.5 g of TSOO/kg BW and 0.25 g of sugar solved in water/kg BW on four different days with a month interval between each. For the elaboration of 250 g of tomato sauces, an amount of 500 g of tomato was used; therefore, the intervention with raw tomatoes was 500 g per 70 kg BW. The amount of sugar used in the control group was assessed in order to give the same amount of kilocalories from carbohydrates in all four intervention groups, around 17.5 g each. All subjects fasted for more than 8 h before each intervention. The day of the intervention, blood samples were collected into plasma ethylenediaminetetraacetic acid (EDTA) tubes at baseline (0 h) and 6 h following the test meal intake. Blood samples were immediately centrifuged after collection (1500 *g* for 20 min at 4 °C), and were then aliquoted and stored at −80 °C until analysis. Urine was collected in plastic containers 10–20 min before consuming the intervention (baseline) and after the ingestion (0–6 h). Before the 6th hour post-intervention, participants were instructed to empty their bladders. The urine was aliquoted and store at −80 °C prior to analysis.

The volunteers refrained from consuming other foods during the 6 h after intervention, while remaining in the clinical ward to avoid the possibility of eating or drinking.

### 2.3. Tomato Sauce Analysis

A commercial tomato variety (*Lycopersicum esculentum L.*) suitable for tomato sauce elaboration (“Smooth tomato”) was used for the study. Tomato sauces were processed at the Torribera campus at the University of Barcelona (Barcelona, Spain) following a standardized procedure. Five percent of refined olive oil was added to the TSOO and the same amount of water was added to the TS in order to standardize the concentration of tomato compounds ingested at each intervention. Both tomato sauces were kept in the refrigerator until served to the subjects in order to avoid any detrimental effect. Tomatoes and tomato sauces were served at room temperature. The phenolic and carotenoid profiles of RT and tomato sauces were determined using an ultra-high pressure liquid chromatography tandem mass spectrometry (UHPLC-MS/MS) and a high pressure liquid chromatography with ultraviolet detection (HPLC-UV), respectively, as previously reported by Vallverdú-Queralt *et al.* [18,19].

### 2.4. Dietary Assessment

Before each intervention, the subjects were asked to fill out a 24-h food recall to assess their compliance with the prescribed diet. Total energy, macronutrient, and micronutrient intake were calculated using the Food Processor Nutrition and Fitness Software (esha Research, Salem, OR, USA). To evaluate any possible adverse effects from the interventions, the dietitian administered a checklist that included mouth symptoms, bloating, fullness or indigestion, altered bowel habit and any other diet-related symptom the day after each intervention.

### 2.5. Measure of Compliance

Tomato phenolic metabolites were determined at baseline in urine spot samples sauces using liquid chromatography coupled to tandem mass spectrometry (LC-MS/MS) as previously reported by Martínez-Huélamo *et al.* [20].

### 2.6. Clinical Measurements

Routine analyses were performed at the CORE laboratory of our Institution that fulfills all required quality criteria. In brief, blood parameters analyzed were the following: blood glucose by glucose oxydase method, total cholesterol and triglycerides by enzymatic procedures; high density lipoprotein (HDL) cholesterol after precipitation with phosphotungstic acid and magnesium chloride all performed in a clinical chemistry analyzer Advia 2400 from Siemens Healthcare (Siemens, Tarrytown, NJ, USA), all reagents were provided by the instrument manufacturer; serum folic acid was measured by an automated electrochemiluminescence immunoassay system Advia-Centaur, Siemens (Siemens, Tarrytown, NJ, USA) with reagents provided by the instrument manufacturer; and insulin by a customized Human Multi Analyte Profiling assay (Human MAP, Rules Based Medicine Inc., Austin, TX, USA).

### 2.7. Peripheral Blood Mononuclear Cells (PBMC) Immunophenotyping

PBMC were isolated from whole blood by density gradient centrifugation with the Ficoll-Hypaque (Pharmacia Biotech, Uppsala, Sweden). The expression of adhesion molecules was analyzed on PBMC surface via double direct immunofluorescence using commercial monoclonal antibodies following the manufacturer’s instructions. The adhesion molecules analyzed were: lymphocyte function-associated antigen-1 (LFA-1) (CD11a; Bender MedSystems, Burlingame, CA, USA), macrophage-1 antigen (or integrin αMβ2) (Mac-1) (CD11b/CD18; Bender MedSystems, Burlingame, CA, USA), very late activation antigen-4 (VLA-4) (CD49d;Cytogmos, Barcelona, Spain), Sialyl-Lewis X (SLex) (CD15s; Beckman Coulter, Fullerton, CA, USA) and CD40 (Caltag Laboratories, Burlingame, CA, USA) for lymphocytes and monocytes, and CD36 (Beckman Coulter Inc., Fullerton, CA, USA) and CCR2 (R&D Systems, Minneapolis, MN, USA) were only measured in monocytes. T-lymphocytes and monocytes were identified separately using anti-CD2 and anti-CD14 (Caltag Laboratories, Burlingame, CA, USA) monoclonal antibodies, respectively. Cell counting (10,000 and 3500 events for the T-lymphocyte and the monocyte regions, respectively) and fluorescence analysis were performed in a FACSCalibur Flow Cytometer (Becton Dickinson, San Jose, CA, USA) using the CellQuestPro software (version 3.3, BD Biosciences). Results are expressed as median fluorescent intensity (MFI) in arbitrary units (AU).

### 2.8. Soluble Inflammatory Markers

The concentration of soluble adhesion molecules is determined using a Human Cytokine Plex (Bio-Rad, Hercules, CA, USA) according to the manufacturer’s instructions. The molecules determined are: Intercellular Adhesion Molecule-1 (ICAM-1), Vascular Cell Adhesion Molecule-1 (VCAM-1), Monocyte Chemoattractant Protein-1 (MCP-1), Interleukin-1α (IL-1α), IL-6, IL-10 and IL-18. Plates were analyzed on a Luminex 100™ instrument (Luminex, Austin, TX, USA) using Bio-Plex Manager TM Software (Bio-Rad, Hercules, CA, USA). Concentrations are obtained by standard calibration curves. Results are shown in pg/mL, except for VCAM-1 and ICAM-1 that are expressed in ng/mL. All measurements are performed in duplicate.

### 2.9. Statistical Analyses

Values are expressed as means ± standard deviation (SD), unless otherwise indicated. The dietary composition was analyzed through a one-factor analysis of variance (ANOVA) for mean comparison since all variables followed a normal distribution according to the results of the Kolmogorov–Smirnov test. To analyze the changes within each treatment a Student’s *t* test for paired samples was performed between the data obtained before and after each intervention. To compare the differences of the changes in outcome variables between the interventions an ANOVA for repeated measures with the Bonferroni post-hoc test was used. All analyses were repeated with adjustment for sex. Differences were considered significant at *p* < 0.05. Statistical analysis was performed using the SPSS Statistical Analysis System (version 18.0; SPSS Inc, Chicago, IL, USA).

## 3. Results

### 3.1. Participants

Forty-three healthy subjects were initially included in the study, but three withdrew before completing it (one because of illness not related to the study and two because of work-related matters). Therefore, 40 subjects (19 men and 21 women) were included in the study with a mean age of 28 ± 11 years old and a mean BMI of 23.30 ± 3.86 kg/m^2^. The participants did not report any adverse effect.

### 3.2. Carotenoids and Phenolics of Tomatoes and Tomato Sauces

The carotenoid and phenolic composition of tomatoes and tomato sauces administered to the subjects was analyzed in order to know how processing affects the contents of these compounds (Table 1). The mean total carotenoids administered was 2067 µg per fresh weight (FW) kg of body weight in the RT intervention, 1346 µg FW/kg in the TS intervention, and 1647 µg FW/kg in the TSOO intervention. The major carotenoid found in the three products was *beta*-carotene followed by lycopene with six-fold lower concentrations. The mean total polyphenols administered was 791 µg FW/kg in the RT intervention, 569 µg FW/kg and 531 µg FW/kg in the TS and the TSOO interventions, respectively. The olive oil added to the TSOO was also analyzed and, as expected, no detectable phenolic and carotenoid compounds were detected.

### 3.3. Energy and Nutrient Intake

According to the 24-h food recall questionnaires, no significant differences were observed in the estimated energy and nutrient intake between intervention groups (Table 2). The estimated intake of polyphenols was approximately 820 mg/day in a Mediterranean population [21], therefore, the intake of 15 to 30 mg/day reported by the participants can be considered negligible and we assumed that the washout diet was correctly followed. In addition, as another measure of compliance, tomato polyphenol metabolites were determined at baseline in urine spot samples, and none was classified as non-compliant (data not shown).

### 3.4. Biochemical Parameters

The overall postprandial responses in blood pressure (BP) and blood parameters after the intervention meals are shown in Table 3. All values were within the reference intervals. The changes observed in the control intervention have been assumed to occur due to the time effect. Significant changes were observed in the lipid profile. Total cholesterol concentrations decreased after the three tomato interventions compared to baseline (*p* < 0.001, all values). Otherwise, low density lipoproteins (LDL)-c concentrations were reduced in the three tomato interventions, although the differences did not attain statistical significance compared to the control (*p* = 0.184). HDL-c concentrations also were increased in these three interventions, but the total-c/HDL ratio only significantly decreased after the TSOO intervention. Plasma triglyceride concentrations decreased after the three tomato interventions compared to baseline. Plasma triglyceride concentrations decreased significantly at six hours after the three tomato interventions compared to baseline (*p* = 0.001 for RT, *p* < 0.001 for TS and *p* = 0.019 for TSOO) but not after the control intervention, this reduction was significantly different between TS and TSOO (*p* = 0.028) diminishing by 33% and 18%, respectively. Plasma folic acid concentrations increased significantly after the three tomato meals (*p* < 0.001) and differed from the control (*p* = 0.001, 0.043, 0.000 for RT, TS and TSOO, respectively).

### 3.5. Adhesion Molecules Expression on PBMCs

Changes in adhesion molecules expression in PBMCs are shown in Table 4. No differences were observed among the baseline data of the four groups of intervention. No changes were observed after interventions in T-lymphocytes except for the significant decrease in LFA-1 expression after the TSOO intervention (*p* = 0.012) and a significant increase after control intervention (*p* = 0.008). In LFA-1, we also found significant differences between TSOO and control (*p* = 0.010). In respect to monocitary adhesion molecule expression, changes in VLA-4 expression were significantly lower after TSOO than after TS (*p* = 0.002). In addition, the expression of SLex did not changed after TSOO, RT and control interventions, but significantly increased after TS (*p* = 0.007). In the same way, CD40 expression showed an increase after TS compared to RT, TSOO and control interventions (*p* = 0.001), whereas CD36 expression showed a significant decrease after TSOO intervention compared to other interventions (*p* = 0.049).

### 3.6. Circulating Inflammatory Biomarkers

The changes in circulating inflammatory markers are shown in Table 5. Compared to baseline, plasma concentration of MCP-1 decreased significantly after the three tomato interventions (*p* = 0.001, 0.013, <0.001 for RT, TS and TSOO, respectively), IL-10 increased after the RT, TS and TSOO interventions (*p* = 0.003, 0.001, 0.020 for RT, TS and TSOO, respectively) and IL-18 was significantly lower after the two sauces interventions (*p* = 0.006 and 0.011 for TS and TSOO, respectively), whereas the concentrations of IL-6 and VCAM-1 only decreased significantly after the TSOO intervention (*p* = 0.006 and 0.020, respectively). Otherwise, the plasma concentrations of the other molecules evaluated remained constant throughout the study. No changes were observed when adjusted for sex.

## 4. Discussion

In this feeding trial performed in 40 healthy subjects, we found that food matrix modified the short term effects of a single tomato intake on lipid profile by decreasing plasma total and ratio total cholesterol/HDL-c. In addition, a single tomato intake decreased some inflammatory biomarker concentrations such as LFA-1, IL-6, IL-18, MCP-1 and VCAM-1, and increased plasma IL-10 concentrations. Moreover, the findings observed demonstrate that tomato sauce in addition to a lipid matrix (refined olive oil) enhanced the effects of tomato intake on the CV system, since the effects of TSOO on plasma inflammatory biomarker concentrations were greater than those of RT and TS.

Diet is a mixture of multiple nutrients that profoundly affects many CVD risk factors [22] and has been recognized to play an important role in the prevention and treatment of atherosclerosis, nowadays considered a low-grade inflammatory disease [23]. Several epidemiological studies have ascribed beneficial anti-inflammatory effects to tomato products. However, published data are unclear, probably due to the fact that the anti-inflammatory effects of tomato products may be affected by industry and/or cooking processing, as well as composition. To date, few human feeding trials have been conducted to study the effect of processed tomato intake in different matrixes on CVD risk factors [4,24,25,26,27]. Besides, in most studies on tomato and tomato products, lycopene was assumed to be responsible for the positive health effects. However, it has been postulated that the decreased risk for developing CVD was more strongly associated with tomato intake with all its components than with isolated lycopene intake [27], suggesting that other compounds may also be involved in the protective cardiovascular effects of tomato. In fact, it should be taken into account that tomato products contain a great variety of compounds including micronutrients such as folic acid, vitamins C, and E, fiber, carotenoids, potassium, magnesium and polyphenols. In addition, the beneficial effects derived from tomato intake could be due, at least in part, to the synergistic effects of these bioactive compounds.

Nutrient bioavailability from dietary sources depends on several factors including the breakup of the food matrix, cooking processes and the presence or addition of lipids or other substances. Moreover, nutrients may interact between them or with other dietary components during digestion, changing their bioavailability. Tomato products’ processing usually involves heat and/or homogenization, both of which can break up the matrix and release and degrade compounds. Several studies have found that heat treatment may decrease the concentration of some micronutrients such as ascorbic acid, total phenolics, lycopene and antioxidant capacity [28] but, at same time, other studies have proved that tomato processing may also increase the bioavailability of certain other bioactive compounds such as lycopene or phenolics [24,29,30]. Besides, during industrial or domestic tomato processing oil is usually added. Since carotenoids are fat-soluble, adding small quantities of fat or oil to the meal may improve their bioavailability [31,32] and even the composition of the oil used may affect antioxidant activity [4,25]. In this setting, we decided to use a homemade tomato sauce instead of commercial preparations in order to allow direct extrapolation of our findings to everyday culinary practices. Thus, we examined the effect of the traditional processing of tomato sauce with refined olive oil, a type of oil that does not contain carotenoids or polyphenols. Therefore, the changes observed may be attributed to differences in the bioavailability of tomato compounds (raw tomato, tomato sauce without oil and tomato sauce with refined olive oil) rather than to the addition of olive oil polyphenols. On the other hand, the dose of active compounds such as carotenoids and polyphenols administered was higher in RT than in the sauces because of processing losses.

In the current study, we also included a control intervention in order to discard changes occurring due to postprandial physiological processes. In this way, changes observed in all interventions including the control cannot be attributed to tomato consumption. Thus, the decrease observed in systolic and diastolic BP after all interventions cannot be attributed to the tomato intake. Despite several mid- and long-term studies have reported beneficial effects on BP [10,27,33] and glucose metabolism [28,31] after antioxidant-rich diets, we did not observe this effect in a postprandial state. In this way, we hypothesize that a single intake of tomato is not enough to obtain the beneficial effects of long term consumption.

Postprandial lipemia is strongly associated with the risk of development of atherosclerotic lesions. It is characterized by the raised levels of triglyceride-rich lipoproteins [34] and is induced by fat meals. In our study, we observed a decrease in triglycerides after the three tomato interventions and, as expected, this change was mitigated after the consumption of TSOO. Moreover, after control intervention, postprandial triglycerides levels returned to baseline, confirming beneficial effect of tomato consumption on postprandial triglyceride plasma concentration. Some studies [30], but not all also observed a reduction in triglycerides after tomato sauce intake, but only when the sauce was prepared with olive oil, and not when sunflower oil was used. Similar to triglycerides, total cholesterol and LDL-cholesterol showed significantly lower concentrations at six hours after the three interventions but not after the control. Regarding cholesterol fractions HDL was found to increase significantly only after TSOO intervention and LDL diminish significantly after TS intake. Since postprandial lipemia is considered a pro-inflammatory process these results are of great importance since systemic inflammation is the basis of the onset and development of atherosclerosis.

Along this line, the interaction of T-lymphocytes and monocytes with the endothelium through adhesion molecules is a crucial event in atheroma plaque formation [23]. Soluble forms of endothelial adhesion molecules found in plasma are considered as an index of endothelial activation and even a biomarker of atherosclerosis [35]. Accordingly, the reduction in IL-6 and VCAM-1 concentrations after a single intake of TSOO (16.9% and 10.4%, respectively) may be due to an inhibited endothelial activation through the inhibition of transcription factors, such as nuclear factor κB, via an activation of a family of inhibitors called IκBs (inhibitors of κB) [23]. Similar results were obtained in another intervention study in which 500 mL a day of *n*-3 PUFA-enriched tomato juice or plain tomato juice were administered to healthy women during 15 days; serum VCAM-1 and ICAM-1 concentrations decreased significantly by 24% and 47%, respectively [36]. Further, Burton-Freeman *et al.* [37] observed that tomatoes reduced postprandial elevation of IL-6 concentrations and concluded that this response could be attributed, at least in part, to the antioxidant effects of tomatoes altering cellular redox status. We also observed a decrease in MCP-1 concentrations after the three interventions with tomato. However, there is only one study in healthy humans evaluating the effects of a tomato product (Mediterranean vegetable soup) on MCP-1. In this study, a decrease in MCP-1 concentrations was found after 14-day intervention [38]. Likewise, we observed a decrease in IL-18 although it only attains statistical significance after the consumption of the two sauces. In contrast, concentrations of IL-10 in plasma increased but only achieved significance after the intake of TS and RT. Interleukin expression are also regulated by the genes involved in the inflammatory response via changes in expression of NF-κB [23].

To our knowledge this is the first study regarding tomato consumption and IL-18 and IL-10 concentrations. Thus, the intake of tomato products may reduce the oxidant and pro-inflammatory effect of meals in the postprandial situation.

Our study has some limitations. Firstly, the study population was healthy and, therefore, the effects observed cannot be extrapolated to high cardiovascular risk populations. Secondly, the acute effects of tomato product consumption may not represent the long-term effects of its consumption. Third, we used a solution of sugar in the control group. Although the number of calories from carbohydrates was similar in the four groups, we cannot exclude a certain acute inflammatory effect of the sugar added [39]. However, our study also has major strengths, such as the study design (cross-over trial including washout periods between interventions), the monitoring of the intake and the compliance, that was excellent according to the results of the analysis of polyphenols in urine samples.

## 5. Conclusions

In conclusion, the results of this study indicate that the intake of tomatoes and tomato sauces, especially tomato sauce enriched with refined olive oil, may regulate the lipid profile and soluble inflammatory biomarkers related to the onset and progression of atherosclerosis. However, confirmatory research focused upon diets containing tomatoes, tomato-based foods and tomato phytochemicals are needed to elucidate the long-term effects of tomato products on health.

## Figures and Tables

**Table 1:** Carotenoids and polyphenols contained in each intervention.

Compounds	Mean ± SD OA * (µg/g)			
	RT	TS	TSOO	Control
**Carotenoids**				
lutein	0.20 ± 0.01	0.38 ± 0.02	0.45 ± 0.10	ND
*alpha*-carotene	0.15 ± 0.04	0.73 ± 0.03	1.06 ± 0.07	ND
*beta*-carotene	246 ± 142	323 ± 179	355 ± 194	ND
*Z*-lycopene isomers ^1^	5.53 ± 1.51	12.4 ± 3.6	27.1 ± 7.3	ND
*E*-lycopene	36.7 ± 8.6	39.3 ± 5.2	77.5 ± 11.9	ND
Total carotenoids	289 ± 106	376 ± 139	461± 150	
Total carotenoids OA (mg) ^2^	144 ± 26.7	94.2 ± 34.9	115 ± 37.5	
**Polyphenols**				
Flavonoids				
Flavanones	103 ± 15.5	146 ± 26.4	135 ± 28.5	ND
Flavanols	4.87 ± 0.29	6.91 ± 0.21	7.75 ± 0.31	ND
Non-flavonoids				
Phenolic acids	2.47 ± 0.12	5.66 ± 0.07	5.38 ± 0.13	ND
Total polyphenols	110 ± 9.98	159 ± 19.1	148 ± 11.9	ND
Total polyphenols OA (mg) ^2^	55.4 ± 8.32	39.8 ± 5.17	37.2 ± 4.09	

Values are means ± SD. RT, raw tomato; TS, olive oil free tomato sauce; TSOO, refined olive oil tomato sauce; OA, Orally administered; ND, Not detected. ^1^ (Z)-lycopene includes all (Z)-isomers ((5Z)-lycopene, (9Z)-lycopene, and (13Z)-lycopene); ^2^ Corresponding to the dose orally administered per 70 kg of body weight.

**Table 2:** Energy, macronutrient and micronutrient intake of the participants before each intervention (24-h food recall).

Parameters	Mean ± SD				*p* ^1^
	RT	TS	TSOO	Control	
Energy (Kcal)	1789 ± 72	1936 ± 101	1865 ± 77	1891 ± 200	0.299
Protein (g)	98.0 ± 5.3	103.0 ± 5.8	105.0 ± 5.3	95.0 ± 16.8	0.438
Carbohydrates (g)	194 ± 11	215 ± 14	199 ± 12	200 ± 47	0.397
Dietary fiber (g)	8.93 ± 0.97	9.33 ± 0.87	7.77 ± 0.95	10.6 ± 3.56	0.652
Total fat (g)	66.2 ± 4.0	71.2 ± 5.4	70.0 ± 4.1	64.2 ± 11.5	0.508
SFA (g)	27.7 ± 1.8	28.0 ± 2.3	27.3 ± 1.7	26.8 ± 5.4	0.619
MUFA (g)	22.4 ± 1.6	25.0 ± 2.1	24.8 ± 1.6	24.7 ± 5.2	0.265
PUFA (g)	10.1 ± 0.9	11.4 ± 1.2	11.6 ± 1.0	11.8 ± 1.7	0.431
Trans fatty acids (g)	1.51 ± 0.27	1.4 ± 0.23	2.3 ± 0.48	1.3 ± 0.79	0.383
Cholesterol (mg)	384 ± 32	351 ± 30	382 ± 30	328 ± 88	0.770
Vitamins					
Pro-vitamin A (RE)	36.8 ± 5.7	32.0 ± 4.3	36.3 ± 7.3	44.2 ± 8.9	0.412
Vitamin A (RE)	448 ± 33	428 ± 41	434 ± 48	493 ± 93	0.803
Vitamin B1 (mg)	1.83 ± 0.11	2.14 ± 0.17	2.00 ± 0.14	2.18 ± 0.60	0.136
Vitamin B2 (mg)	2.06 ± 0.11	2.06 ± 0.14	2.10 ± 0.10	2.13 ± 0.40	0.482
Vitamin B3 (mg)	20.6 ± 1.4	25.1 ± 1.5	23.9 ± 1.4	21.2 ± 6.1	0.148
Vitamin B6 (mg)	1.31 ± 0.13	1.42 ± 0.15	1.39 ± 0.12	1.37 ± 0.28	0.440
Vitamin B12 (μg)	5.54 ± 0.64	5.5 ± 0.68	6.44 ± 0.86	4.62 ± 2.28	0.590
Vitamin C (mg)	8.30 ± 1.40	7.41 ± 1.66	8.81 ± 1.33	9.65 ± 2.14	0.455
Vitamin E (μg)	6.65 ± 0.65	6.30 ± 0.92	6.60 ± 0.59	6.50 ± 0.83	0.968
Minerals					
Folate (μg)	350 ± 28	377 ± 43	358 ± 30	404 ± 141	0.730
Calcium (mg)	1040 ± 72	893 ± 84	957 ± 57	837 ± 238	0.759
Magnesium (mg)	211 ± 13	252 ± 37	208 ± 10	209 ± 72	0.467
Phosphorus (mg)	1494 ± 75	1440 ± 95	1494 ± 63	1370 ± 261	0.960
Potassium (mg)	1729 ± 101	1829 ± 106	1860 ± 96	1876 ± 283	0.385
Selenium (μg)	129 ± 8	138 ± 9	139 ± 9	128 ± 37	0.559
Sodium (mg)	2564 ± 207	2737 ± 269	2505 ± 199	2540 ± 1140	0.912
Zinc (mg)	11.1 ± 0.8	11.4 ± 0.8	12.0 ± 0.8	11.1 ± 2.3	0.739
Total polyphenols (mg)	27.2 ± 3.1	26.4 ± 3.7	26.1 ± 5.1	24.0 ± 7.7	0.766

Values are means ± SD. RT, raw tomatoes; TS, olive oil free tomato sauce; TSOO, refined olive oil tomato sauce; SFA, saturated fatty acid; RE, retinol equivalent. SFA, saturated fatty acid; MUFA, monounsaturated fatty acid; PUFA, polyunsaturated fatty acid. ^1^ Data analyzed by one factor analysis of variance (ANOVA) for repeated measures.

**Table 3:** Changes in blood pressure and biochemical parameters at baseline and six hours after the four interventions.

		RT		TS		TSOO		Control		*p* ^3^
		Mean ± SD ^1^	Mean Differences (95% CI) ^2^	Mean ± SD ^1^	Mean Differences (95% CI) ^2^	Mean ± SD ^1^	Mean Differences (95% CI) ^2^	Mean ± SD ^1^	Mean Differences (95% CI) ^2^	
SBP (mm Hg)	Before	120 ± 13	−5.67 (−8.90 to −2.43)	117 ± 13	−1.95 (−5.38 to 1.47)	120 ± 12	−5.57 (−8.49 to −2.64)	118 ± 9	−3.44 (−6.63 to −0.25)	0.135
	After	115 ± 11 **		115 ± 10		115 ± 12 **		115 ± 9 **		
DBP (mm Hg)	Before	72.0 ± 9.5	−2.21 (−4.51 to 0.10)	73.0 ± 7.8	−1.46 (−3.65 to 0.73)	73.0 ± 8.1	−2.92 (−5.18 to −0.66)	71.0 ± 6.8	−1.98 (−3.63 to −0.34)	0.689
	After	70.0 ± 9.3		72.0 ± 9.6		70.0 ± 9.0 *		69.0 ± 8.0 **		
Total cholesterol (mg/dL)	Before	167 ± 28	−6.53 (−9.99 to −3.07) ^a^	170 ± 29	−8.49 (−12.0 to −4.96) ^a^	167 ± 23	−9.03 (−13.5 to −4.55) ^a^	168 ± 16	−1.57 (−6.69 to 3.55) ^b^	0.005
	After	160 ± 28 **		162 ± 26 **		158 ± 22 **		167 ± 13		
HDLc (mg/dL)	Before	52.0 ± 11.2	0.42 (−1.28 to 2.12)	52.0 ± 11.8	1.09 (−2.45 to 4.63)	52.0 ± 11.3	2.36 (0.19 to 4.54)	50.0 ± 12.7	−0.03 (−4.19 to 4.14)	0.404
	After	53.0 ± 11.1		53.0 ± 12.4		55.0 ± 13.3 *		50.0 ± 11.9		
LDLc (mg/dL)	Before	95.0 ± 19.6	−2.88 (−6.30 to 0.53)	96.0 ± 22.6	−4.31 (−7.80 to −0.83)	95.0 ± 20.5	−2.00 (−5.41 to 1.41)	95.0 ± 13.9	−0.37 (−3.48 to 2.74)	0.184
	After	92.0 ± 19.0		92.0 ± 18.2 *		93.0 ± 18.9		95.0 ± 11.8		
Triglycerides (mg/dL)	Before	84.0 ± 54.7	−22.6 (−34.5 to −10.7) ^a,b^	86.0 ± 42.0	−28.4 (−41.6 to −15.2) ^a^	83.0 ± 36.5	−15.3 (−22.4 to −8.12) ^b^	86.0 ± 23.8	−2.73 (−13.1 to 7.67) ^c^	0.002
	After	62.0 ± 34.8 **		57.0 ± 22.7 **		68.0 ± 31.7 **		83.0 ± 31.6		
Ratio Total-c/HDL	Before	3.30 ± 0.90	−0.17 (−0.31 to −0.02) ^a^	3.40 ± 0.90	−0.24 (−0.46 to −0.02) ^a^	3.30 ± 0.90	−0.28 (−0.49 to −0.07) ^a^	3.60 ± 0.90	−0.04 (−0.34 to 0.26) ^b^	0.034
	After	3.20 ± 0.90 *		3.20 ± 0.80 *		3.10 ± 0.90 **		3.50 ± 0.90		
Folic acid (serum)	Before	6.50 ± 3.00	1.91 (1.31 to 2.51) ^a^	6.00 ± 2.10	1.86 (1.26 to 2.46) ^a^	6.10 ± 2.30	1.94 (1.51 to 2.37) ^a^	6.50 ± 2.00	0.18 (−0.36 to 0.71) ^b^	<0.001
(ng/mL)	After	8.40 ± 2.80 **		7.90 ± 2.70 **		8.00 ± 2.00 **		6.70 ± 2.30		

Results expressed as ^1^ mean ± SD and ^2^ mean differences (95% CI) between after and before each intervention. ^3^ *p* value of the ANOVA for repeated-measures from the differences between interventions. Different superscript letters in a row show significant difference between interventions by Bonferroni *post-hoc test* (*p* < 0.05). * Significant differences (*p* < 0.05) and ** significant differences (*p* < 0.01) between time in the intervention, measured by the Student’s *t* test for paired samples. RT, Raw tomato; TS, olive oil free tomato sauce; TSOO, refined olive oil enriched tomato sauce; SBP, systolic blood pressure; DBP, diastolic blood pressure; Total-c, total cholesterol.

**Table 4:** Expression of adhesion molecules on PBMC at baseline and 6 hours after the four interventions.

		RT		TS		TSOO		Control		*p* ^3^
		Mean ± SD ^1^	Mean Differences (95% CI)^2^	Mean ± SD ^1^	Mean Differences (95% CI) ^2^	Mean ± SD ^1^	Mean Differences (95% CI) ^2^	Mean ± SD ^1^	Mean Differences (95% CI) ^2^	
T-lymphocytes (MFI)		AU								
LFA-1	Before	143 ± 58	4.40 (−6.80 to 15.5) ^a^	146 ± 59	1.60 (−7.70 to 10.8) ^a^	144 ± 50	−9.60 (−17.0 to −2.20) ^b^	141 ± 22	9.70 (2.70 to 16.60) ^c^	0.010
	After	147 ± 60		148 ± 54		135 ± 43*		150 ± 27 *		
Mac-1	Before	141 ± 59	−0.20 (−16.8 to 16.3)	144 ± 51	−6.40 (−21.9 to 9.10)	143 ± 69	−4.80 (−24.4 to 14.8)	147 ± 73	0.30 (−30.2 to 30.7)	0.814
	After	141 ± 55		137 ± 37		138 ± 45		147 ± 23		
VLA-4	Before	60.0 ± 15.8	1.10 (−2.70 to 4.90)	60.0 ± 11.4	2.20 (−1.20 to 5.50)	62.0 ± 14.9	1.90 (−1.40 to 5.20)	58.0 ± 14.9	1.70 (−14.1 to 17.6)	0.983
	After	61.0± 18.3		62.0 ± 10.3		63.0 ± 14.7		60.0 ± 38.2		
SLex	Before	234 ± 104	−2.50 (−24.4 to 19.4)	237 ± 109	−6.80 (−36.2 to 22.6)	237 ± 137	−1.70 (−41.3 to 37.8)	242 ± 30.9	−0.90 (−17.4 to 15.7)	0.925
	After	231 ± 113		231 ± 108		235 ± 109		241 ± 14.6		
CD40	Before	138 ± 36	7.00 (−10.1 to 24.0)	139 ± 42	5.20 (−8.60 to 19.0)	138 ± 36	1.10 (−12.5 to 14.8)	141 ± 22	3.70 (−1.50 to 8.80)	0.879
	After	145 ± 47		144 ± 36		139 ± 44		144 ± 19		
Monocytes (MFI)		AU								
LFA-1	Before	83.0 ± 27.5	2.20 (−6.30 to 10.7)	80.0 ± 21.2	3.50 (−1.60 to 8.60)	82.0 ± 15.6	2.10 (−2.80 to 6.90)	83.0 ± 8.00	2.90 (−1.10 to 7.00)	0.561
	After	85.0 ± 26.2		84.0 ± 16.5		84.0 ± 20.1		86.0 ± 14.1		
Mac-1	Before	57.0 ± 11.0	0.50 (−2.90 to 3.80)	57.0 ± 10.7	0.60 (−3.20 to 4.30)	58.0 ± 11.7	−0.20 (−3.20 to 2.80)	58.0 ± 12.0	0.50 (−5.70 to 6.60)	0.343
	After	57.0 ± 12.3		57.0 ± 11.9		57.0 ± 10.2		58.0 ± 21.1		
VLA-4	Before	45.0 ± 12.9	1.00 (−2.30 to 4.20) ^a,b^	43.0 ± 7.90	2.20 (−0.30 to 4.80) ^a^	44.0 ± 9.40	−0.90 (−3.00 to 1.30) ^b^	40.0 ± 29.3	3.00 (−9.70 to 15.7) ^a^	0.030
	After	46.0 ± 13.4		46.0 ± 8.20		43.0 ± 9.70		43.0 ± 9.20		
SLex	Before	84.0 ± 27.1	−0.70 (−6.10 to 4.70) ^a^	84.0 ± 18.3	3.70 (−0.90 to 8.30) ^b^	87.0 ± 25.6	0.10 (−6.30 to 6.60) ^a^	85.0 ± 12.7	0.50 (−2.70 to 3.70) ^a^	0.007
	After	84.0 ± 24.9		87.0 ± 19.0		87.0 ± 21.6		86.0 ± 8.60		
CD40	Before	51.0 ± 16.3	−1.80 (−5.70 to 2.10) ^a^	47.0 ± 11.6	2.70 (−0.70 to 6.00) ^b^	48.0 ± 11.7	−0.90 (−5.50 to 3.70) ^a^	49.0 ± 13.6	0.30 (−2.90 to 3.40) ^a^	0.001
	After	49.0 ± 17.3		50.0 ± 13.0		47.0 ± 15.2		49.0 ± 8.80		
CD36	Before	52.0 ± 10.6	0.40 (−3.60 to 4.50) ^a^	51.0 ± 13.2	−0.30 (−4.50 to 3.80) ^a^	54.0 ± 20.2	−6.20 (−13.2 to −0.01) ^b^	51.0 ± 24.9	−0.10 (−22.7 to 22.4) ^a^	0.042
	After	53.0 ± 12.3		51.0 ± 10.0		47.0 ± 18.0 *		51.0 ± 25.6		
CCR2	Before	435 ± 108	−8.30 (−61.2 to 44.7)	437 ± 149	−10.6 (−53.0 to 31.7)	439 ± 138	−12.0 (−47.0 to 23.0)	440 ± 295	−13.9 (−171 to 143)	0.998
	After	427 ± 141		427 ± 164		427 ± 115		426 ± 524		

Results expressed as ^1^ mean ± SD and ^2^ mean differences (95% CI) between after and before each intervention. ^3^ *p* value of the repeated-measures ANOVA from the differences between interventions. Different superscript letters in a row show significant difference between interventions by Bonferroni *post-hoc test* (*p* < 0.05). * Significant differences (*p* < 0.05) between time in the intervention, measured by the Student’s *t* test for paired samples. PBMC, Peripheral blood mononuclear cells; RT, Raw tomato; TS, olive oil free tomato sauce; TSOO, refined olive oil enriched tomato sauce. Results were expressed as median fluorescent intensity (MFI), in arbitrary units (AU). VLA-4, very late activation antigen-4; LFA-1, lymphocyte function-associated antigen-1; Mac-1, chemoattractant activation-dependent molecule; SLe^x^, anti-Sialyl-Lewis X.

**Table 5:** Concentrations of circulating inflammatory biomarkers in plasma at baseline and 6 hours after the three interventions.

		RT		TS		TSOO		Control		*p* ^3^
		Mean ± SD ^1^	Mean Differences (95% CI)^2^	Mean ± SD ^1^	Mean Differences (95% CI) ^2^	Mean ± SD ^1^	Mean Differences (95% CI) ^2^	Mean ± SD ^1^	Mean Differences (95% CI) ^2^	
IL-1α (pg/mL)	Before	0.79 ± 0.46	−0.02 (−0.13 to 0.08)	0.76 ± 0.43	−0.08 (−0.18 to 0.03)	0.74 ± 0.38	−0.04 (−0.14 to 0.05)	0.79 ± 0.41	−0.05 (−0.27 to 0.18)	0.856
	After	0.76 ± 0.45		0.69 ± 0.32		0.69 ± 0.36		0.75 ± 0.25		
IL-6 (pg/mL)	Before	6.28 ± 2.45	−0.30 (−0.92 to 0.32) ^a,b^	6.35 ± 2.81	−0.55 (−1.31 to 0.22) ^a,b^	6.47 ± 3.16	−1.09 (−1.84 to −0.34) ^a^	7.09 ± 1.31	0.76 (−0.11 to 1.63) ^b^	0.029
	After	5.99 ± 2.49		5.81 ± 2.83		5.38 ± 2.60 **		6.33 ± 2.28		
IL-10 (pg/mL)	Before	2.29 ± 0.60	0.20 (0.07 to 0.32) ^a^	2.18 ± 0.53	0.18 (0.07 to 0.28) ^a^	2.25 ± 0.54	0.15 (0.02 to 0.28) ^a^	2.33 ± 0.57	−0.21 (−0.39 to −0.23) ^b^	0.014
	After	2.49 ± 0.61 *		2.36 ± 0.62 **		2.40 ± 0.56 **		2.12 ± 0.49 *		
IL-18 (pg/mL)	Before	120 ± 49	−3.92 (−13.4 to 5.61)	124 ± 45	−9.42 (−15.9 to −2.90)	129 ± 61	−12.7 (−22.4 to −3.08)	116 ± 37	3.42 (−8.91 to 15.7)	0.456
	After	116 ± 49		115 ± 47 **		116 ± 55*		120 ± 29		
MCP-1 (pg/mL)	Before	32.2 ± 19.1	−6.12 (−9.71 to −2.53) ^a^	32.0 ± 17.6	−5.27 (−9.37 to −1.17) ^a^	32.7 ± 18.7	−6.82 (−10.0 to −3.60) ^a^	30.0 ± 8.8	3.20 (0.77 to 5.73) ^b^	0.001
	After	26.1 ± 14.7 **		26.8 ± 16.0 **		25.9 ± 13.6 **		33.3 ± 6.5		
ICAM-1 (ng/mL)	Before	202 ± 88	9.60 (−5.85 to 25.0)	207 ± 89	4.52 (−13.1 to 22.1)	204 ± 81	−9.51 (−20.3 to 1.35)	212 ± 52.6	0.41 (−10.3 to 11.1)	0.244
	After	212 ± 92		211 ± 100		194 ± 100		212 ± 53.6		
VCAM-1(ng/mL)	Before	367 ± 144	−17.8 (−45.5 to 9.98) ^a^	354 ± 125	−14.4 (−47.1 to 18.1) ^a^	375 ± 135	−38.8 (−71.3 to −6.47) ^b^	380 ± 89	2.62 (−42.1 to 47.4) ^a^	0.042
	After	349 ± 115		339 ± 136		336 ± 118 *		382 ± 133		

Results expressed as ^1^ mean ± SD and ^2^ mean differences (95% CI) between after and before each intervention. ^3^ *p* value of the repeated-measures ANOVA from the differences between interventions. Different superscript letters in a row show *significant difference between* interventions by Bonferroni *post-hoc test* (*p* < 0.05). * Significant differences (*p* < 0.05) and ** significant differences (*p* < 0.01) between time in the intervention, measured by the Student’s *t* test for paired samples. RT, Raw tomato; TS, olive oil free tomato sauce; TSOO, refined olive oil enriched tomato sauce; MCP-1, Monocyte chemotactic protein-1; ICAM-1, intercellular *adhesion Molecule-1*; VCAM-1, vascular cell adhesion molecule-1.

## Supplementary Materials

Supplementary materials are available online at http://www.mdpi.com/2072-6643/8/3/170/s1.
