# Supplementary material for: Tomato Sauce Enriched with Olive Oil Exerts Greater Effects on Cardiovascular Disease Risk Factors than Raw Tomato and Tomato Sauce: A Randomized Trial
Source: Nutrients. 2016 Mar 16;8(3):170. doi: 10.3390/nu8030170 (PMC4808898; doi:10.3390/nu8030170)
Supplement: Supplementary file 1 [file nutrients-08-00170-s001.docx]

Supplementary Materials: Tomato Sauce Enriched with Olive Oil Exerts Greater Effects on Cardiovascular Disease Risk Factors than Raw Tomato and Tomato Sauce: A Randomized Trial

Palmira Valderas-Martinez, Gemma Chiva-Blanch, Rosa Casas, Sara Arranz,
Miriam Martínez-Huélamo, Mireia Urpi-Sarda, Xavier Torrado, Dolores Corella,
Rosa M. Lamuela-Raventós and Ramon Estruch


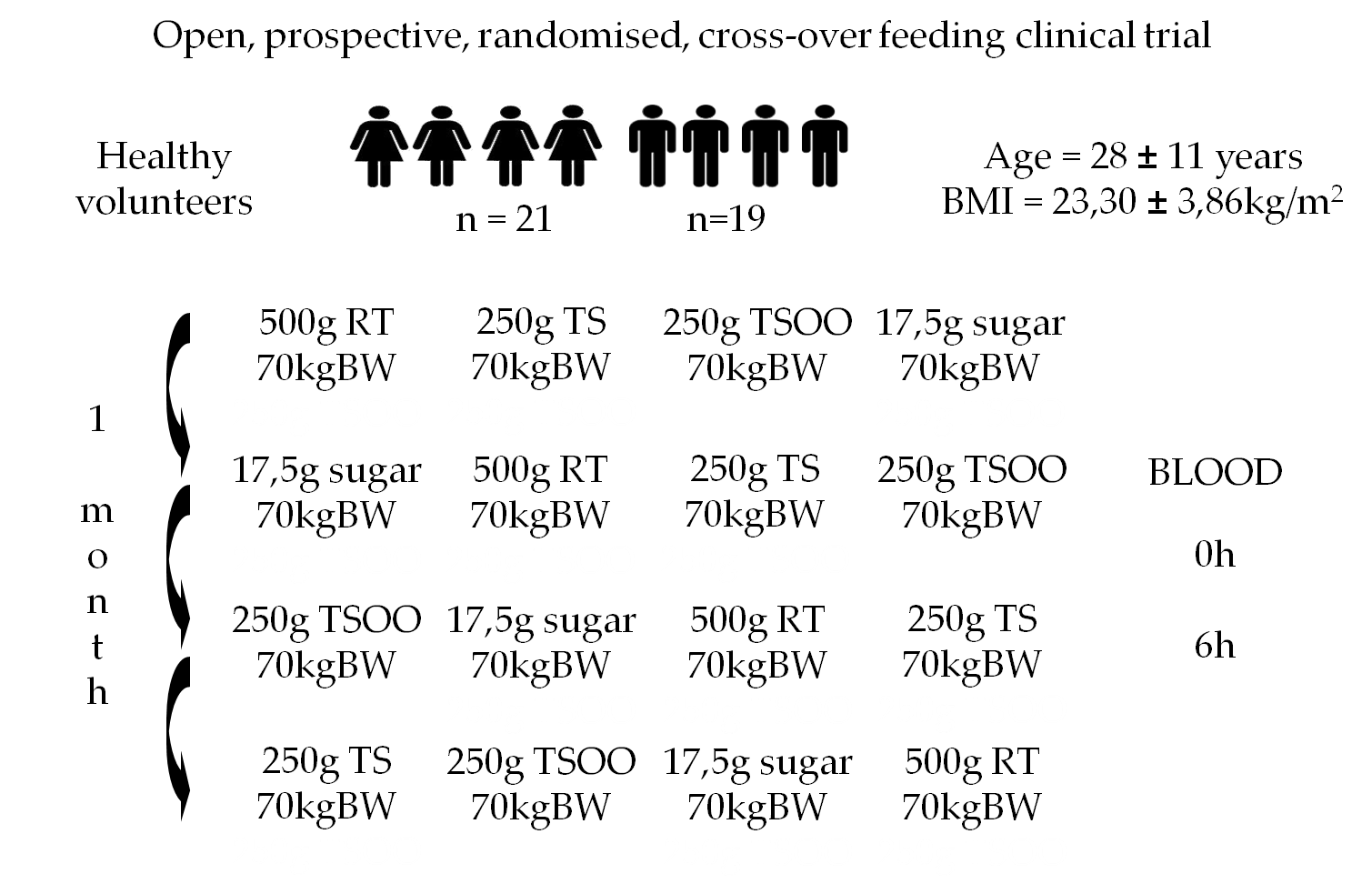


**Figure S1**. Study design.
